# Case Report: Pulsed field ablation of an oblique inferoparaseptal accessory pathway

**DOI:** 10.3389/fcvm.2026.1801903

**Published:** 2026-04-14

**Authors:** Hans Ebbinghaus, Norbert Klein

**Affiliations:** Department of Cardiology, Hospital Sankt Georg, Leipzig, Germany

**Keywords:** accessory pathway, preexcitation, pulse field ablation, SVT (supraventricular tachycardia), Wolff-Parkinson-White

## Abstract

**Background:**

Radiofrequency ablation (RFA) remains the gold standard for Wolff-Parkinson-White (WPW) syndrome with ∼94% acute success, ∼6% recurrence. Pulsed field ablation (PFA) shows promise for complex accessory pathways (APs) but lacks data on oblique courses.

**Case presentation:**

A 36-year-old endurance athlete presented with incidental right paraseptal pre-excitation before methylphenidate initiation. He reported self-terminating palpitations (∼200 bpm) during moderate cycling. Electrophysiological study revealed an oblique inferoparaseptal AP with right atrial insertion (inferior interatrial septum/CS roof, Kent potential) and left inferoparaseptal ventricular exit (earliest retrograde activation mid-CS 5/6). Due to right coronary artery proximity, focal PFA via Centauri™ system (Galvanize) achieved complete CS ostium/roof block. Post-procedure ventricular pacing showed retrograde decremental conduction. At 3 months, he reported no tachycardias/palpitations.

**Discussion/conclusion:**

This case demonstrates focal PFA's feasibility for an underrepresented oblique inferoparaseptal AP amid complex CS/CSMC anatomy, avoiding RFA's coronary injury risk. While early PFA series report >99% acute success, randomized long-term data vs. RFA are needed. PFA represents a promising investigational approach for anatomically challenging APs.

## Background

Wolff-Parkinson-White (WPW) syndrome is characterized by atrioventricular accessory pathways (AP) that may cause supraventricular tachycardia and, rarely, sudden cardiac death. Catheter-based radiofrequency ablation (RFA) is established as first-line curative therapy and is considered the current gold standard. Modern systematic reviews and large registries report acute success rates around 94%, recurrence rates of approximately 6%, and complication rates near 1% (1–3). In contrast, pulsed field ablation (PFA) for APs and WPW is at an early stage of clinical adoption. Current data consist of small single-center case series and a larger prospective single-arm trial in paroxysmal supraventricular tachycardia (PSVT) using a combined focal PFA/RF catheter (6, 7). These early experiences suggest high acute success and a favorable safety profile but lack randomized comparisons with RFA and robust long-term follow-up (6, 7).

This report describes PFA of an oblique inferoparaseptal AP with right atrial insertion and left inferoparaseptal ventricular exit, illustrating how coronary sinus (CS)-related anatomy and myocardial sleeves can shape mapping strategy and energy.

## What's New?

This case demonstrates the feasibility and acute efficacy of focal PFA for an anatomically complex oblique inferoparaseptal accessory pathway with right atrial insertion and left ventricular exit-a substrate underrepresented in current PFA series.

## Case presentation

A 36-year-old male endurance athlete was referred for electrophysiological study after incidental pre-excitation detection prior to methylphenidate initiation. He reported episodes of abrupt palpitations with heart rates up to approximately 200 bpm during moderate cycling, lasting about 20 min and self-terminating, in some cases with vagal maneuvers. He remained asymptomatic during competitive sports. There was no history of syncope, presyncope, or documented tachycardia ECG. Family history was negative for sudden cardiac death.

**Figure 1:**
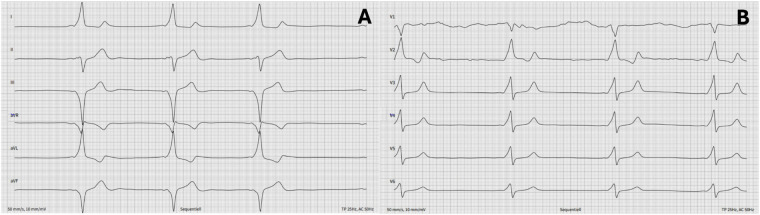
12-lead ECG of a 36-year-old man showing pre-excitation with negative delta wave in V1, absent in inferior leads, and positive in V3-consistent with right paraseptal accessory pathway per pambrun et al. (24).

Physical examination, laboratory tests, and echocardiography were unremarkable, showing a structurally normal heart. Baseline ECG demonstrated manifest pre-excitation consistent with a right-sided paraseptal accessory pathway per the Pambrun algorithm (8) (Figure 1). Ablation was planned due to athlete status.

During the electrophysiological study, multipolar catheters were positioned in the right atrium and right ventricle (4-pole), coronary sinus (10-pole), along with an Advisor™ HD Grid Mapping Catheter, Sensor Enabled™ (Abbott; 8 F, 16-electrode 4 × 4 grid, 3 mm spacing), and a contact force-sensing ablation catheter. Electroanatomical mapping was performed using the EnSite Precision™ system (Abbott). At baseline, the earliest ventricular activation and most pronounced pre-excitation were recorded at the proximal coronary sinus (CS 9/10), suggesting a right-sided AP, with a long anterograde refractory period of approximately 800 ms. Given his status as an athlete, a shared decision was made to proceed with ablation of the AP.

**Figure 2:**
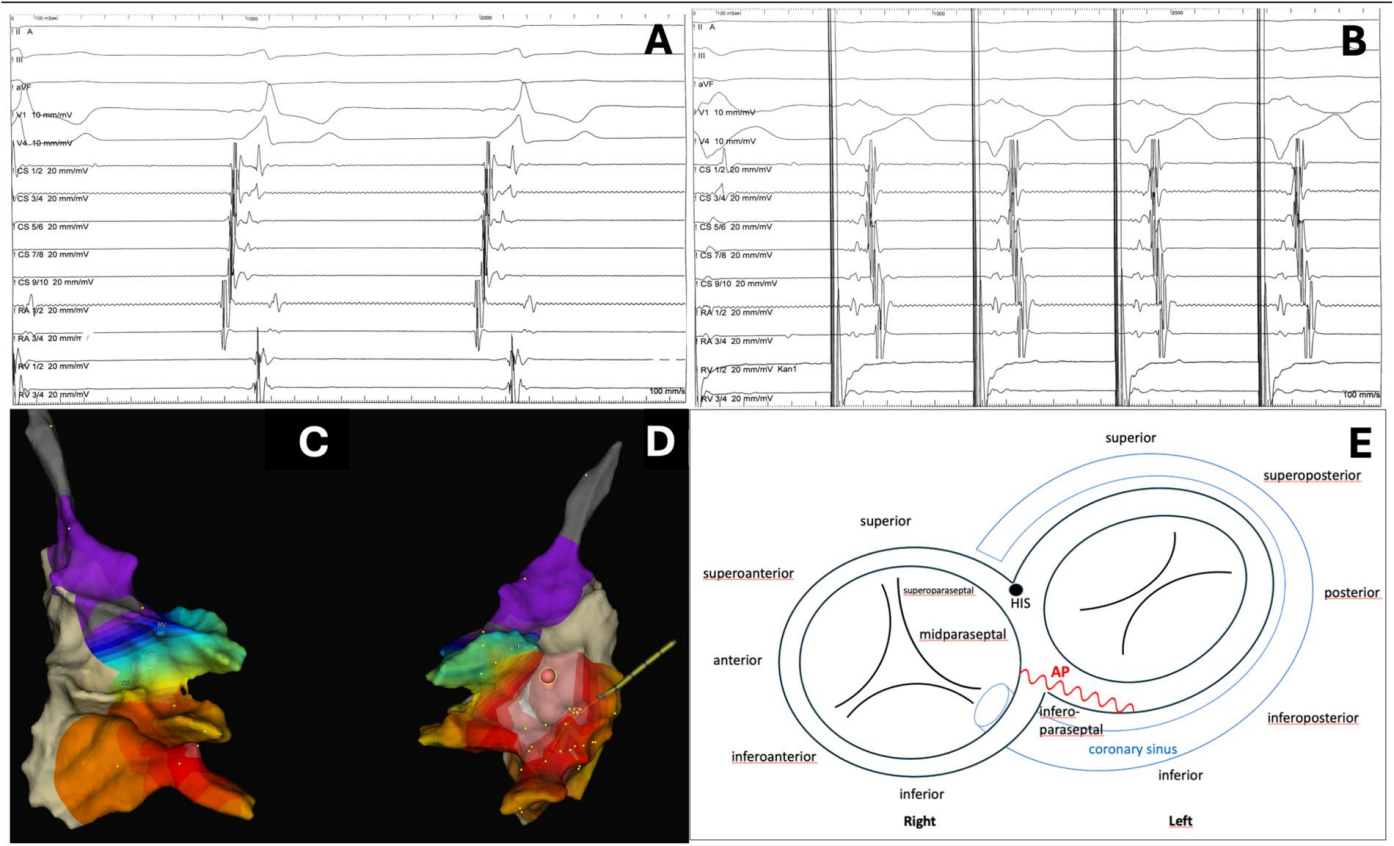
Electrophysiology study. **(A)** Baseline sinus rhythm: earliest ventricular activation and maximal pre-excitation at proximal CS (9/10), suggesting a right-sided AP. **(B)** RV pacing: eccentric atrial activation, earliest at mid-CS (5/6), indicating a left-sided inferoparaseptal AP. **(C,D)** HD-Grid mapping (EnSite Precision): earliest atrial activation at the inferior interatrial septum near the CS roof (atrial insertion); **(C)** right anterior oblique (RAO) view, **(D)** left anterior oblique (LAO) view. **(E)** Schematic valvular plane with inferoparaseptal oblique AP (red tortuous line): atrial insertion at the inferior interatrial septum above the CS roof, ventricular exit inferoparaseptal on the left side. HIS: His bundle.

During ventricular pacing, a retrogradely conducting, non-decremental pathway was documented, with earliest atrial activation at CS 5/6 (mid-CS), indicating eccentric atrial activation consistent with a left-sided inferoparaseptal AP. Fluoroscopic control confirmed correct coronary sinus catheter position. CS aneurysm was ruled out by angiography. Right atrial mapping demonstrated earliest atrial activation at the inferior interatrial septum near the CS roof, suggesting the atrial insertion of the pathway in this region. Overall, the findings were consistent with an oblique inferoparaseptal accessory pathway, with right-sided atrial insertion at the inferior interatrial septum (above the coronary sinus roof), as indicated by the presence of a Kent bundle potential at the successful ablation site, and a more left-sided inferoparaseptal ventricular exit (Figure 2). No supraventricular tachycardia was inducible, precluding confirmation of the presence of multiple accessory pathways. Invasive induction of atrial fibrillation and calculation of the shortest preexcited RR interval (SPERRI) were not performed. As the patient was a competitive endurance athlete with manifest ventricular pre-excitation, we followed contemporary guideline recommendations that support an invasive strategy with curative catheter ablation in young patients and athletes with pre-excitation, and therefore proceeded directly to ablation without additional AF-based risk stratification testing.

**Figure 3:**
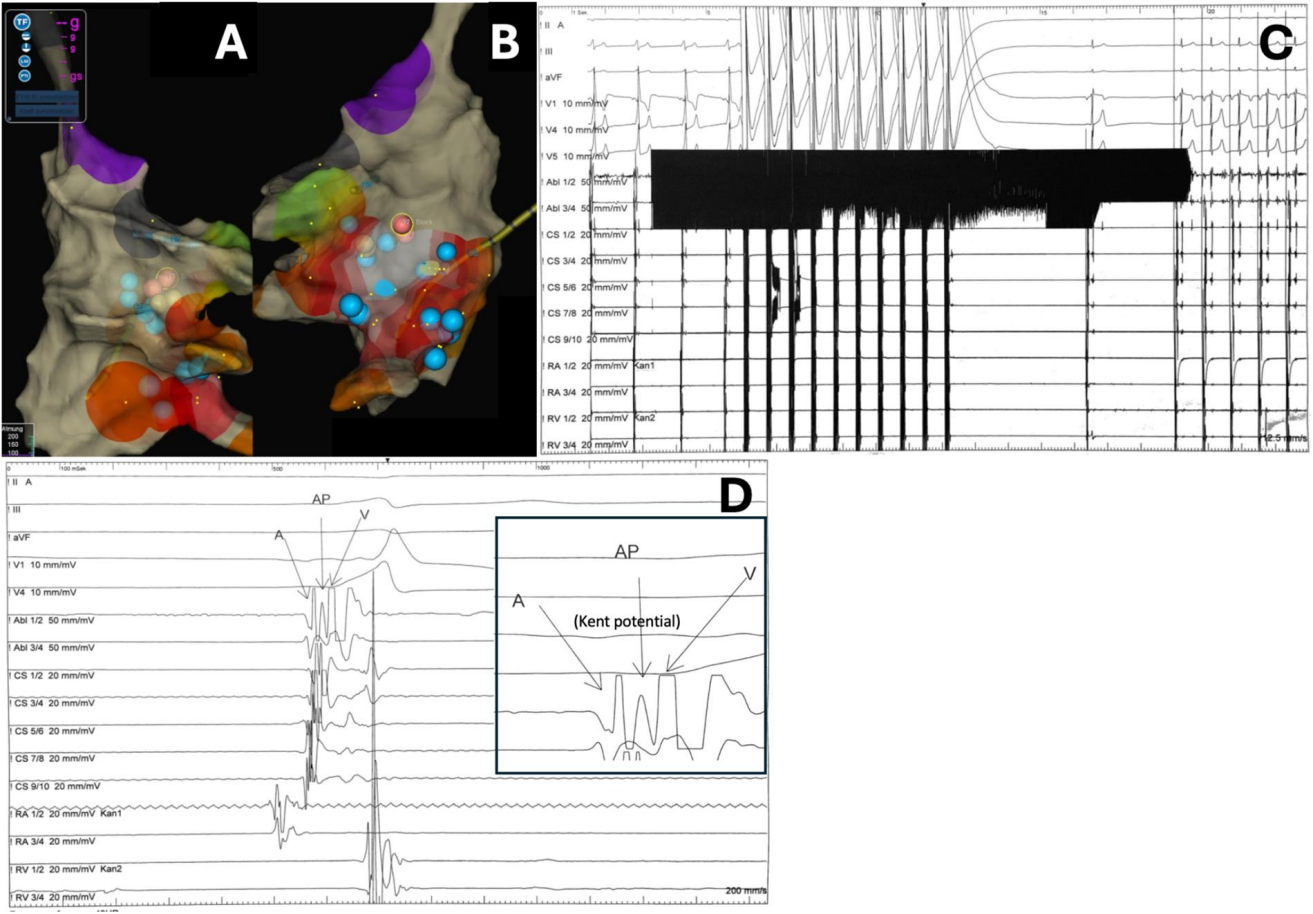
Pulsed field ablation (PFA) and outcome. **(A,B)** Centauri™ PFA system: multiple lesions from the inferior interatrial septum to the CS roof, achieving conduction block at the CS ostium/roof; **(A)** right anterior oblique (RAO) view, **(B)** left anterior oblique (LAO) view. **(C)** Successful AP ablation: no residual pre-excitation; atrial pacing for vagally induced bradycardia. **(D)** Reproducible Kent bundle potential (AP potential) recorded between local atrial and ventricular electrograms at the atrial insertion of an oblique posteroseptal accessory pathway (inferior interatrial septum/CS roof), with termination upon.

**Figure 4:**
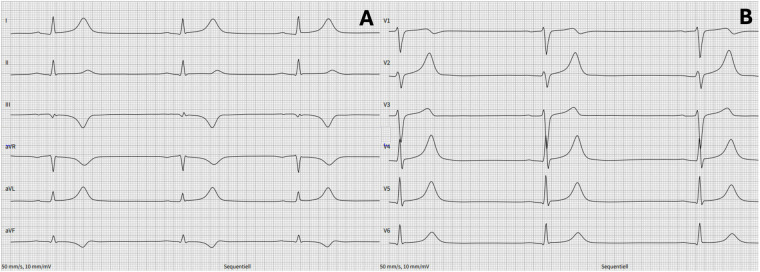
Post-procedure 12-lead ECGs: no pre-excitation evident, notable cardiac memory in III and aVF.

Ablation was performed using a TactiCath™ Sensor Enabled™ contact force ablation catheter (Abbott; 3.5 mm irrigated tip, 2-2-2 mm ring spacing) with the EnSite Precision™ system and TactiSys™ Quartz module. PFA was chosen due to the oblique inferoparaseptal pathway's complex anatomy and its proximity to right coronary artery branches, which increased the potential risk of complications with RFA. PFA was delivered using the Centauri™ PFA system (Galvanize) via a contact force-sensing catheter in a focal, point-by-point fashion. In total, 17 focal applications were delivered from the inferior interatrial septum to the CS roof, targeting the atrial insertion of the oblique inferoparaseptal accessory pathway, with lower energy settings applied in regions closest to the septum and adjacent coronary structures. This strategy achieved complete conduction block at the CS ostium/roof (Figure 3). Given the inferoparaseptal location and potential proximity to right coronary artery branches, we implemented specific safety precautions: continuous ECG and hemodynamic monitoring were performed, we were prepared to perform urgent coronary angiography if ischemic changes occurred, and nitroglycerin was prepared and readily available for immediate administration in case of suspected coronary spasm. Ventricular stimulation revealed subsequent retrograde decremental conduction. At 3 months, the patient reported no tachycardias or palpitations. A postprocedure 12-lead ECG showed complete resolution of pre-excitation with features of cardiac memory in the inferior leads (Figure 4).

## Discussion

RFA remains the reference standard for the treatment of WPW syndrome, with consistently high acute success rates and low complication rates demonstrated in meta-analyses and large registry studies (1–5). PFA has recently emerged as a non-thermal ablation modality that induces preferential myocardial injury while relatively sparing adjacent non-cardiac structures. In a prospective multicenter PSVT study including patients with WPW/AVRT, focal PFA used in combination with RF achieved >99% acute success, with only very low rates of transient AV block and no permanent AV conduction injury (6). Data on “pure” focal PFA for accessory pathway (AP) ablation remain limited; the largest single-center experience reported by Brešković et al. included 14 APs (3 posteroseptal, 3 right free wall, and 8 left-sided) treated exclusively with focal PFA (7). In that series, all pathways were acutely eliminated without complications, and only one recurrence was observed during a median follow-up of 5.5 months. Left-sided APs were preferentially targeted from within the coronary sinus (CS), with 7 of 8 successfully ablated via a CS approach alone (7). Larger PFA series in PSVT include a broad spectrum of AP locations but do not specifically characterize complex oblique pathways with right atrial insertion and left-sided ventricular exit (6). Overall, the available evidence suggests very high acute success rates of focal PFA for AP ablation, very low observed complication rates-including in septal and CS regions and in pediatric cohorts -but also highlights the absence of long-term randomized data comparing PFA with RFA and the limited information available for anatomically complex, oblique, or epicardial CS-related APs (6, 7).

In the present case, the earliest atrial activation during right ventricular pacing was recorded at CS 5/6, a finding not consistent with a straightforward right-sided accessory pathway. This pattern raises several possibilities, including atypical conduction along an oblique pathway course, directional dependence of activation, mapping limitations, or the presence of multiple pathways. Taken together, the findings are most consistent with an oblique inferoparaseptal AP, with atrial insertion on the right side (inferior interatrial septum above the CS ostium) and a more left-sided inferoparaseptal ventricular exit. Such oblique or epicardial courses are well described and may mimic multiple distinct pathways when mapping is incomplete (9–12). The coronary sinus myocardial coat (CSMC) forms extensive muscular connections to both atria and to the ventricle via extensions along the middle cardiac vein and posterior coronary veins (10, 13, 14). These CSMC-ventricular connections underlie paraseptal and left inferior APs and can result in atypical activation patterns, with earliest atrial signals recorded in the mid CS, middle cardiac vein, or CS roof rather than at the expected annular sites. Bridging fibers and epicardial connections may lead to earlier activation in the mid CS than in the proximal CS or produce discordant activation sequences between CS and annular recordings (10, 13, 15). In addition, CS roof APs may demonstrate a proximal atrial insertion during sinus rhythm but a functional retrograde exit in the mid CS during right ventricular pacing, resulting in direction-dependent discrepancies in activation mapping (10, 16). As emphasized by Lebloa and Pascale, paraseptal and inferoparaseptal APs share a complex “crux” anatomy in which closely spaced right-sided, left-sided, and epicardial CS-related courses are associated with lower acute success and higher recurrence rates following RFA, as well as with unexpected earliest activation sites (14). Case reports and small series of oblique or epicardial CS-related APs-including diverticulum-associated pathways-describe earliest activation within the mid-CS or venous aneurysms, with delayed proximal CS or annular recordings; successful ablation often requires targeting epicardial CS or coronary venous structures (10, 11, 13, 17, 18). Ablation of inferoparaseptal pathways using RFA is challenging due to anatomical complexity and proximity to coronary arteries, increasing complication risk. Studies report high acute success rates (up to 98%) for inferoparaseptal APs, yet recurrences and repeat procedures are frequent, particularly right-sided (19, 20). Proximity to coronary arteries poses significant risk: RFA within 2 mm caused injury in up to 50% of cases (21).

Within this context, focal PFA delivery from the CS or tributaries offers a precise strategy for ablating epicardial/CS-related AP segments, potentially reducing thermal injury to coronary arteries or the AV conduction system vs. RFA (5, 7, 14, 17). PFA near coronary arteries often provokes reversible vasospasm-as observed at the CTI (RCA) and mitral isthmus (LCX)-typically resolving within minutes after nitrates, without permanent ischemia (22–27). CS roof-specific spasm reports remain limited; however, anatomical risks warrant precautions for CS-based PFA: careful imaging, nitrate readiness/prophylaxis, and ECG monitoring.

The high acute success of CS-based PFA for left-sided APs reported by Brešković et al. supports this approach in anatomically challenging settings and is consistent with the favorable acute outcome observed in the present case (7).

## Conclusion

This case illustrates that focal PFA can successfully treat an oblique inferoparaseptal AP with right atrial and left inferoparaseptal ventricular insertions amid complex CS/CSMC anatomy, enabling effective ablation while avoiding irreversible coronary artery injury. While early data suggest excellent acute efficacy and a favorable safety profile, PFA for WPW remains investigational pending larger randomized trials and long-term follow-up vs. RFA.

## Data Availability

The original contributions presented in the study are included in the article/Supplementary Material, further inquiries can be directed to the corresponding author.
